# Body fat mobilization in early lactation influences methane production of dairy cows

**DOI:** 10.1038/srep28135

**Published:** 2016-06-16

**Authors:** A. Bielak, M. Derno, A. Tuchscherer, H. M. Hammon, A. Susenbeth, B. Kuhla

**Affiliations:** 1 Leibniz Institute for Farm Animal Biology (FBN), Institute of Nutritional Physiology “Oskar Kellner”, Wilhelm-Stahl-Allee 2, 18196 Dummerstorf, Germany; 2 Leibniz Institute for Farm Animal Biology (FBN), Institute of Genetics and Biometry, Wilhelm-Stahl-Allee 2, 18196 Dummerstorf, Germany; 3 Institute of Animal Nutrition and Physiology, Christian-Albrechts-Universität zu Kiel, Hermann-Rodewald-Straße 9, 24118 Kiel, Germany

## Abstract

Long-chain fatty acids mobilized during early lactation of dairy cows are increasingly used as energy substrate at the expense of acetate. As the synthesis of acetate in the rumen is closely linked to methane (CH_4_) production, we hypothesized that decreased acetate utilization would result in lower ruminal acetate levels and thus CH_4_ production. Twenty heifers were sampled for blood, rumen fluid and milk, and CH_4_ production was measured in respiration chambers in week −4, +5, +13 and +42 relative to first parturition. Based on plasma non-esterified fatty acid (NEFA) concentration determined in week +5, animals were grouped to the ten highest (HM; NEFA > 580 μmol) and ten lowest (LM; NEFA < 580 μmol) mobilizing cows. Dry matter intake (DMI), milk yield and ruminal short-chain fatty acids did not differ between groups, but CH_4_/DMI was lower in HM cows in week +5. There was a negative regression between plasma NEFA and plasma acetate, between plasma NEFA and CH_4_/DMI and between plasma cholecystokinin and CH_4_/DMI in week +5. Our data show for the first time that fat mobilization of the host in early lactation is inversely related with ruminal CH_4_ production and that this effect is not attributed to different DMI.

The fermentation of poly- and monosaccharides in the rumen results in the formation of pyruvate which in turn serves as intermediate substrate for the production of short-chain fatty acids, primarily acetate, propionate and butyrate. Propionate synthesis is a hydrogen consuming process, whereas the conversion of pyruvate to acetate or butyrate is accompanied by the production of hydrogen. Methanogenic microbiota utilize the released hydrogen to reduce CO_2_ yielding in the formation of CH_4_^1^. Accordingly, the ruminal acetate or the (acetate + butyrate) : propionate ratio is highly related with daily CH_4_ emission (g/d) or CH_4_ yield expressed as g per unit of dry matter intake (g/kg DMI)^2^,^3^. In the post-absorptive metabolism of dairy cows, propionate is primarily used for hepatic gluconeogenesis, whereas acetate is activated to form acetyl-CoA serving as the main energy-providing substrate for the host. In lactating cows, acetate and butyrate may also serve as precursors for de novo milk fat (C4 – C16) synthesis by the mammary gland. Thus, CH_4_ and precursors for de novo milk fatty acids arise from the same biochemical pathway in the rumen. Therefore concentrations of milk fatty acids de novo synthesized from acetate, e.g. C6, C8, C10 and C16 or the sum of these saturated fatty acid concentrations are indicators for the level of acetate production in the rumen and were found to be positively related to CH_4_ emission or CH_4_ yield^3^,^4^,^5^.

The (acetate + butyrate) : propionate ratio in the rumen decreases when the diet is supplemented with oilseeds rich in C18 fatty acids, resulting in reduced CH_4_ production^6^. The CH_4_ suppressing effect of dietary long-chain fatty acids may be due to decreased fiber digestibility or reduced DMI^7^. In lactating animals, C18 milk fatty acid concentrations increased at the expense of shorter chain milk fatty acids upon supplementing the diet with oilseeds^6^,^7^. Hence, concentrations of C18 milk fatty acids were found to be negatively related to CH_4_ yield^3^,^4^,^5^. However, neither DMI, feed composition, ruminal short-chain fatty acids nor milk fatty acids alone are sufficient to accurately predict CH_4_ emission^3^, indicating that either the microbial composition or traits of the host may influence methanogenesis in the rumen. Only recently Ricci *et al*.^8^ considered, besides dietary characteristics, the physiological stage of the animal to improve the prediction of CH_4_ emissions.

During the transition from late pregnancy to early lactation, high-yielding dairy cows do not ingest enough feed to meet the nutrient and energy requirements for milk production and consequently enter into negative energy balance. During this time of lactation provision of energy by dietary derived acetate is lowest and as an adaptive response cows mobilize their body fat reserves leading to increased concentrations of circulating long-chain, non-esterified fatty acids (NEFA). These mobilized NEFA are mainly composed of C16 and C18 and are intensively oxidized to allocate energy but also used by the mammary gland for milk fat synthesis^9^. Plasma NEFA concentrations paralleled the concentrations of milk C18, while the concentration of milk fatty acids synthesized from acetate remained unaffected or were inversely associated with plasma NEFA levels until week 7 after parturition^9^. Thus, the increase in plasma NEFA concentrations reduces the level of acetate utilization, at least in the mammary gland, but whether plasma NEFA concentrations are also negatively related to CH_4_ yield remains to be investigated. Herein, we hypothesized that increased body fat mobilization resulting in higher plasma NEFA concentrations would negatively affect metabolic acetate utilization, and as a kind of negative feed-back regulation, acetate production in the rumen and consequently CH_4_ production.

## Results

### Animals and Diets

**Figure 1:**
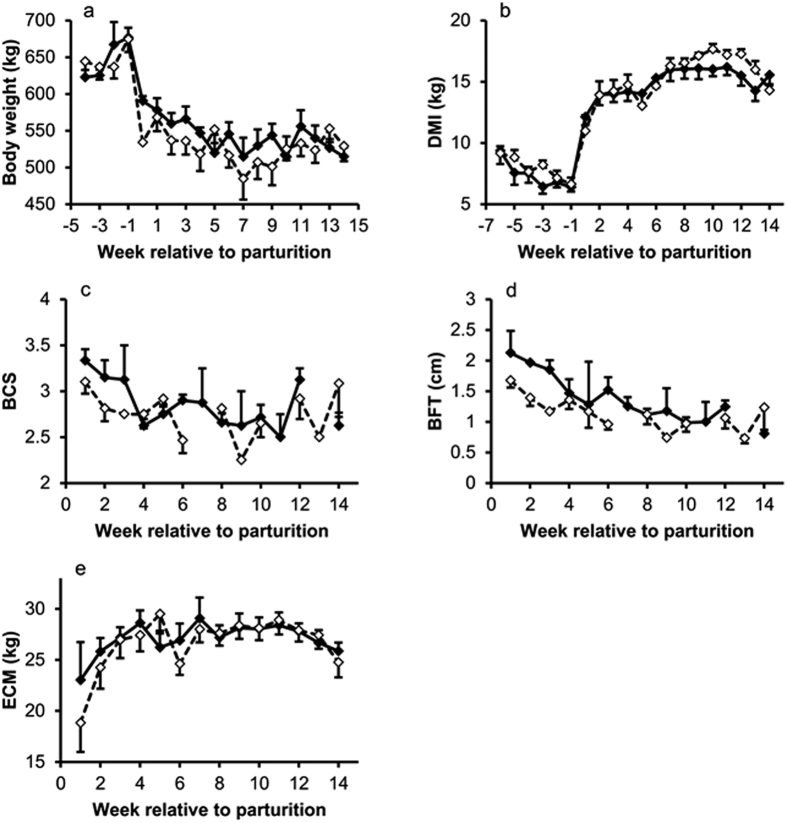
Body weight, DMI, BCS, BFT and ECM of high (solid line; n = 10) and low mobilizing (◊, dashed line; n = 10) cows during the period of first lactation. ANOVA calculated time × group interactions were: (**a**) *P* = 0.18, (**b**) *P* = 0.04, (**c**) *P* = 0.60, (**d**) *P* = 0.02, (**e**) *P* = 0.15. Data are shown as LSM ± SE.

Per design, animals were grouped according to their plasma NEFA concentrations at the time of the respiration chamber measurements in week 5 ± 0.2 of early lactation in which plasma NEFA concentrations amounted to 811.2 ± 61.9 μmol/L for the ten highest (HM; NEFA > 580 μmol) and 379.1 ± 61.9 μmol/L for the ten lowest (LM; NEFA < 580 μmol) mobilizing cows (P < 0.001; Supplemental Fig. 1a). The HM cows had also greater total amounts of plasma NEFA in the time period two weeks before until six weeks after the respiration chamber measurement in week 5 ± 0.2 (*P* = 0.007, Supplemental Fig. 1b), indicating slower increase but longer-lasting fat mobilization compared to LM cows who showed earlier, shorter and less intensive peak concentration far (i.e. 4 weeks) before respiration chamber measurement (702.3 ± 123.7 μmol/L; Supplemental Fig. 1a). The body weight of HM and LM cows ranged from 610 kg to 665 kg 4 weeks before parturition and changed over time (*P* < 0.001), but was not different between groups (Fig. 1a; Supplemental Table S1). From late pregnancy until week 13 post partum (p.p.) body weight declined to 556 ± 112 kg after which it increased again. DMI increased after calving (*P* < 0.001), but without difference between groups at any time point (Fig. 1b; Supplemental Table S1). In week 4 ante partum (a.p.) animals ingested about 7.1 kg of DM per d, which increased to 13.2 kg, 14.5 kg and 15.2 kg in week 5, 13 and 42 p.p., respectively, without difference between groups at times indicated (Supplemental Table S1). Body condition score (BCS) and back fat thickness (BFT) were not different between groups, but changed over time (*P* < 0.001; Fig. 1c,d; Supplemental Table S1). However, the group × time interaction for BFT was significant (*P* = 0.02; Fig. 1d). Precisely, HM cows had a higher BFT at calving (P = 0.007) and a higher loss of back fat until week 5 p.p. (P = 0.006, Fig. 1d).

Energy corrected milk yield (ECM) increased from week 1 until week 4 p.p. and decreased for all cows over time (*P* < 0.001) from 28.8 ± 1.1 kg in week 5 p.p. to 27.6 ± 0.8 kg in week 13 p.p. to 24.8 ± 0.9 kg in week 42 p.p., but again there was no differences between HM and LM cows (Fig. 1e, Supplemental Table S1). Milk fat decreased from week 5 p.p. to week 13 p.p. in both cow groups and was significantly lower in HM compared to LM cows in week 13 p.p. (HM 3.72 and LM 4.34 ± 0.13%; *P* = 0.03; Supplemental Table S1).

Feed digestibility determined in week 6 p.p. was comparable between groups (77.4 ± 0.3% in HM and LM cows; *P* > 0.1). The mean retention time (MRT) in week 6 p.p. amounted to 27.5 ± 0.6 h in HM cows and to 27.0 ± 0.6 h in LM cows without difference (*P* = 0.54) between groups.

### Plasma, Milk and Rumen fluid

**Figure 2:**
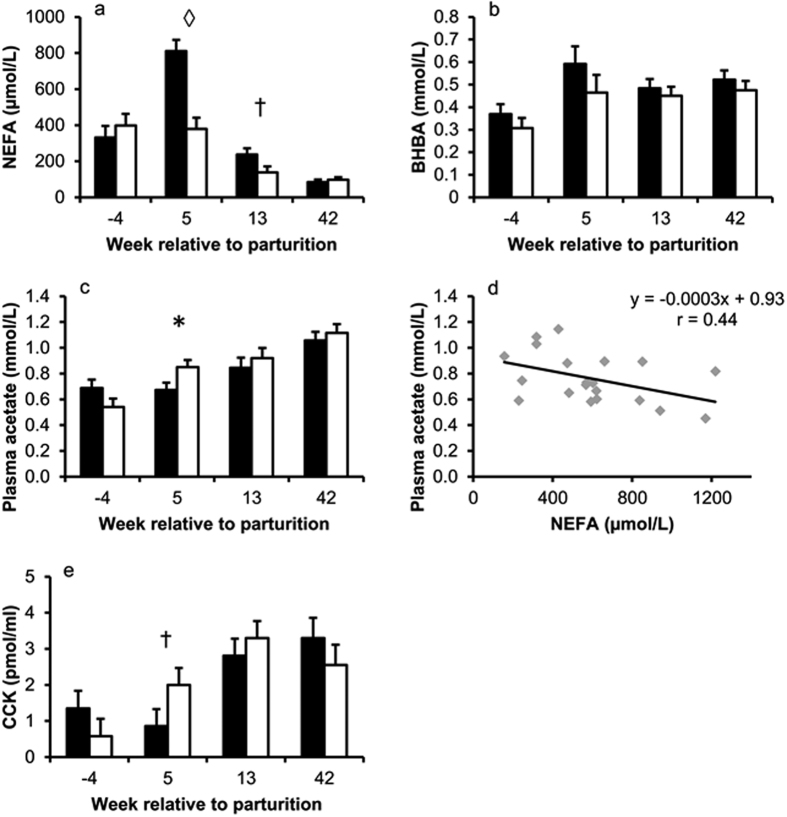
Plasma NEFA, BHBA and acetate concentrations at different time points, and regression between plasma acetate and plasma NEFA concentrations in week 5 p.p. Cows grouped as high mobilizing are marked ■ (n = 10), cows grouped as low mobilizing are marked □ (n = 10). (**a**) ◊ Indicates the time point of grouping the animals. (**b**) Time *P* = 0.01, group *P* = 0.18, time × group *P* = 0.87, ANOVA. (**c**) Time *P* < 0.001, group *P* = 0.32, time × group *P* = 0.13, ANOVA; * indicates *P* = 0.04, Tukey-Test. (**d**) Slope *P* = 0.053, n = 20. (**e**) Time *P* = 0.003, group *P* = 0.95, time × group *P* = 0.12, ANOVA; † indicates *P* = 0.1. Data in bar charts are shown as LSM ± SE.

**Figure 3:**
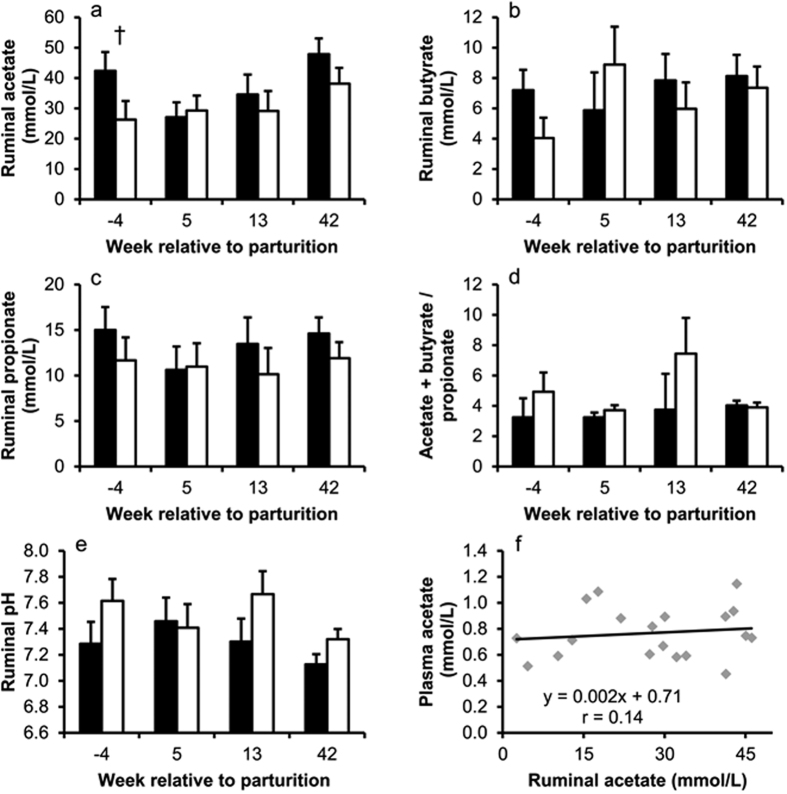
Ruminal variables as group means at the times of respiration chamber measurements in cows with high (■; n = 10) and low (□; n = 10) plasma NEFA concentrations in week 5 p.p. and regression between plasma and ruminal acetate concentrations measured in week 5 p.p. (**a**) Time *P* = 0.045, group *P* = 0.045, time × group *P* = 0.44, ANOVA; † indicates *P* = 0.09; Tukey-Test. (**b**) Time *P* = 0.71, group *P* = 0.55, time × group *P* = 0.61, ANOVA. (**c**) Time *P* = 0.61, group *P* = 0.25, time × group *P* = 0.81, ANOVA. (**d**) Time *P* = 0.17, group *P* = 0.11, time × group *P* = 0.48, ANOVA. (**e**) Time *P* = 0.16, group *P* = 0.12, time × group *P* = 0.57, ANOVA. (**f**) Slope *P* = 0.57. Data in bar charts are shown as LSM ± SE.

Plasma NEFA concentrations were greater in HM than LM cows not only in week 5 (P < 0.001), but still tended to be higher in week 13 after parturition (*P* = 0.1, Fig. 2a). Plasma beta-hydroxybutyrate (BHBA) concentrations increased after parturition (*P* < 0.01), but there were no differences (*P* = 0.18) between groups (Fig. 2b). Plasma acetate concentrations continuously increased over time (*P* < 0.001) and were lower in HM compared to LM cows (0.67 and 0.85 ± 0.06 mmol/L, respectively) in week 5 p.p. (*P* = 0.04; Fig. 2c). Accordingly, plasma NEFA and plasma acetate concentrations showed a significant (*P* < 0.001) inverse relationship in week 5 p.p. (Fig. 2d). Plasma cholecystokinin (CCK) concentrations increased after parturition (*P* < 0.01) and tended to be lower in HM compared to LM cows in week 5 p.p. (*P* = 0.1; Fig. 2e). Ruminal acetate concentrations varied with time (each *P* < 0.05) and pair-wise comparison for each time point revealed that HM cows tended (*P* = 0.087) to have higher rumen acetate concentrations in week 4 a.p. only (Fig. 3a). Ruminal propionate and butyrate, the (acetate + butyrate) : propionate ratio and ruminal pH were not influenced by time or group (Fig. 3b–e). In addition, linear regression between ruminal and plasma acetate was not significant (Fig. 3f; *P* = 0.57).

### Methane, Fat and Carbohydrate Oxidation

**Figure 4:**
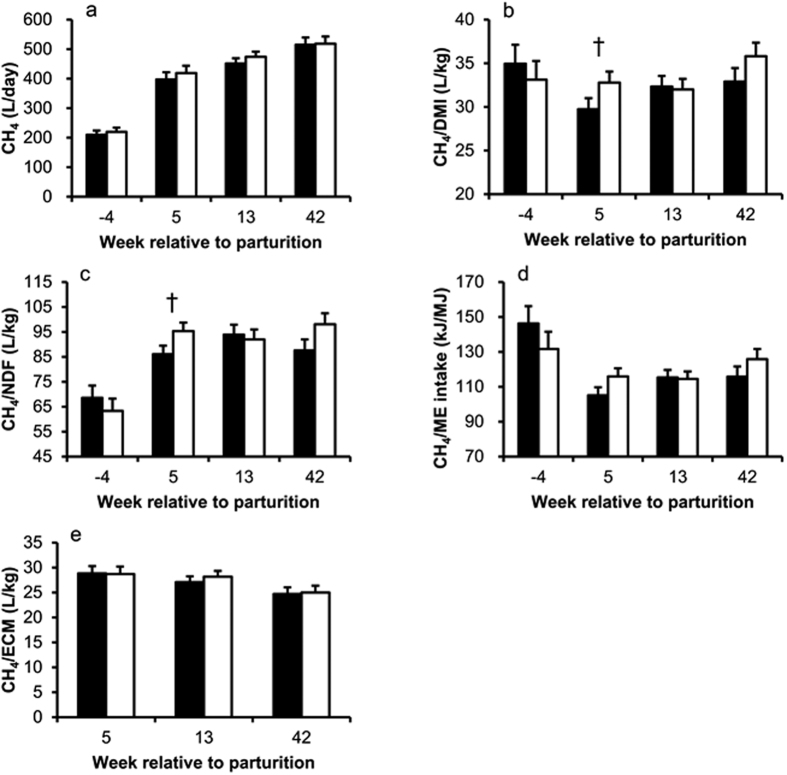
Daily CH_4_ production, CH_4_ per dry matter intake (CH_4_/DMI), CH_4_ per neutral detergent fiber (CH_4_/NDF), CH_4_ per metabolizable energy (ME) intake (CH_4_/ME intake), and CH_4_ per energy corrected milk yield (CH_4_/ECM) in cows with high (■; n = 10) and low (□; n = 10) plasma NEFA concentrations in week 5 p.p. (**a**) Time *P* < 0.001, group *P* = 0.51, time × group *P* = 0.91, ANOVA. (**b**) Time *P* = 0.17, group *P* = 0.54, time × group *P* = 0.14, ANOVA; † indicates *P* = 0.1, Tukey-Test. (**c**) Time *P* < 0.001, group *P* = 0.66, time × group *P* = 0.91, ANOVA; † indicates *P* = 0.08, Tukey-Test. (**d**) Time *P* < 0.001, group *P* = 0.44, time × group *P* = 0.09, ANOVA. (**e**) Time *P* < 0.023, group *P* = 0.82, time × group *P* = 0.14, ANOVA. Data in bar charts are shown as LSM ± SE.

Total CH_4_ production increased over time (*P* < 0.001), but was not different between groups (Fig. 4a) and CH_4_ yield did not change over time (Fig. 4b). However, in week 5 p.p. HM cows tended to have lower CH_4_ emission when related to DMI (29.7 and 32.8 ± 1.3 L/kg, respectively; *P* = 0.1; Fig. 4b) or to neutral detergent fiber (NDF) (86.0 and 95.3 ± 3.5 L/kg, respectively; *P* = 0.08; Fig. 4c) than LM cows. But when CH_4_ emission was related to ME intake, groups did not differ at any time point (Fig. 4d). Methane emission per kilogram ECM decreased over time (*P* < 0.01), but was not different between groups (Fig. 4e).

**Figure 5:**
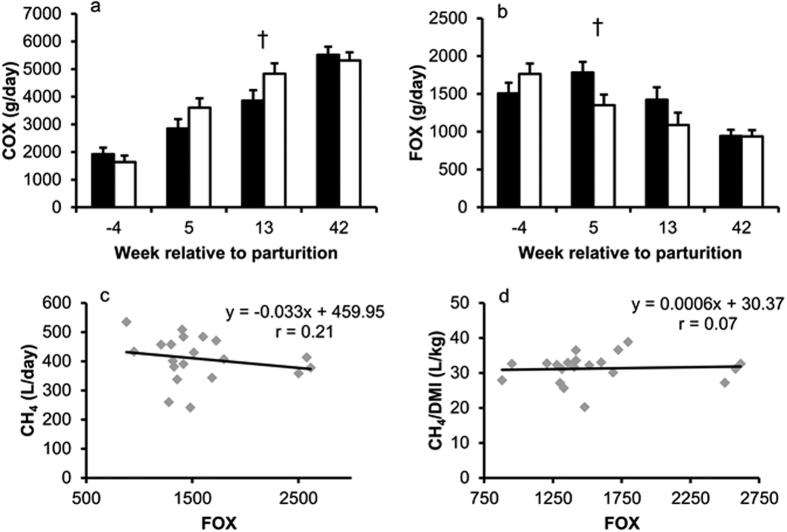
Carbohydrate oxidation (COX) and fat oxidation (FOX) at different time points in cows with high (■; n = 10) and low (□; n = 10) plasma NEFA concentrations in week 5 p.p.; and linear regression of FOX and CH_4_ yield (n = 20). (**a**) Time *P* < 0.001, group *P* = 0.24, time × group *P* = 0.08, ANOVA; † indicates *P* = 0.09; Tukey-Test. (**b**) Time *P* < 0.001, group *P* = 0.14, time × group *P* = 0.22, ANOVA; † indicates *P* = 0.06; Tukey-Test. (**c**) Slope *P* = 0.37. (**d**) Slope *P* = 0.78. Data in bar charts are shown as LSM ± SE.

Net carbohydrate oxidation (COX) continuously increased until week 42 of lactation and tended to be different (*P* = 0.09) between cow groups in week 13, but not in week 5 p.p. (Fig. 5a). After parturition net fat oxidation (FOX) decreased over time in both groups (*P* < 0.001; Fig. 5b). The pair-wise comparison in week 5 p.p. showed that HM cows tended to have a higher fat oxidation than LM cows (1783 and 1352 ± 140 g/d, respectively; *P* = 0.058; Fig. 5a), but there was no significant regression between net FOX and CH_4_ (*P* = 0.37) or CH_4_/DMI (*P* = 0.78), respectively (Fig. 5c,d).

**Figure 6:**
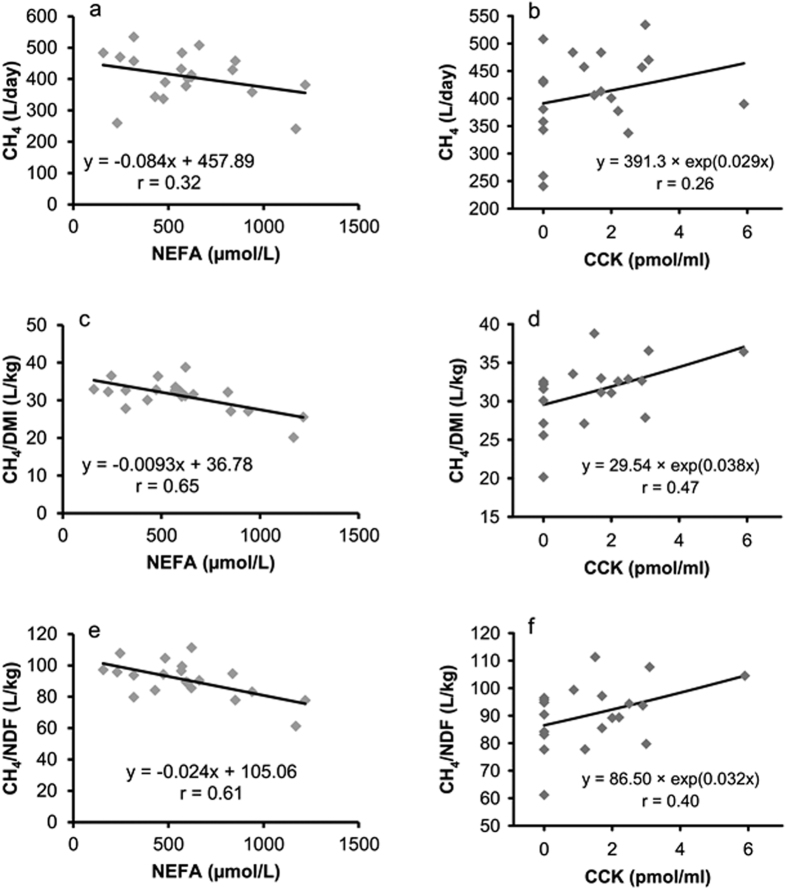
Linear regression between plasma NEFA concentration and daily CH_4_ production either expressed as L/d, L/kg DMI, or L/kg NDF, respectively and 2 parametrical exponential regression (y = a × e^(bx)^)between plasma CCK concentrations and CH_4_ production (n = 20). (**a**) Slope *P* = 0.17. (**b**) *P* < 0.0001 for a; *P* = 0.28 for b. (**c**) Slope *P* = 0.002. (**d**) *P* < 0.0001 for a; *P* = 0.03 for b. (**e**) Slope *P* = 0.005. (**f**) *P* < 0.0001 for a; *P* = 0.07 for b.

Linear regression between CH_4_ production and plasma NEFA concentration in week 5 p.p. was not significant (Fig. 6a; *P* = 0.17), whereas we found an inverse relationship between CH_4_ yield expressed as CH_4_/DMI (*P* = 0.002) or CH_4_/NDF (*P* = 0.005) with plasma NEFA concentrations of individual cows in week 5 p.p. (Fig. 6c,e). These regressions were not evident in week −4, +13 and +42 relative to parturition (data not shown). Furthermore, we were not able to detect any significant relationship between CH_4_ production and ruminal acetate concentrations (Supplemental Fig. 2).

The best fitted curve for a relationship between CCK and CH_4_, CH_4_/DMI or CH_4_/NDF was a two parametrical exponential function, but the coefficients of determination only reached R^2^ = 0.06, R^2^ = 0.23 and R^2^ = 0.17, respectively (Fig. 6b,d,f).

## Discussion

We hypothesized that increased body fat mobilization in early lactation associated with reduced acetate utilization of the host would negatively affect ruminal acetate concentration and CH_4_ production. To examine this hypothesis we retrospectively grouped the cows according to their plasma NEFA concentrations in early lactation (i.e. week 5) to high and low mobilizing cows and determined CH_4_ production at different physiological stages throughout the lactation cycle. Both HM and LM cows had a continuous increase in daily CH_4_ production from one month before parturition to lactation week 42. This course does not parallel the biphasic course of CH_4_ production predicted by mid-infrared spectra validated by the SF_6_ technique^10^. A decrease in CH_4_ production in late lactation however, may be initiated by a diet change often applied during that time, while our cows were kept on the same diet throughout lactation. Alterations in CH_4_ emission during the lactation cycle can be due to the increasing rumen capacity during early lactation, accompanied by a passage rate that is decoupled from DMI, as well as changes in the digestibility of feed^11^. In agreement with their greater plasma NEFA concentrations, HM cows had the greater loss of back fat thickness during the first five weeks of lactation. Recent studies showed that elevated plasma NEFA concentrations around parturition are utilized for about 40% of milk fat synthesis during early lactation^12^,^13^. Other authors discovered that cows with greater fat mobilization produce higher milk fat contents and thus a higher ECM in early lactation^14^. In our study, ECM and milk fat content did not differ between groups in week 5 p.p. Reasons for this may be the difference in the dynamic of fat mobilization with LM cows showing an early but short and less intensive peak NEFA concentration while HM cows had slowly increasing but longer-lasting fat mobilization (Supplemental Fig. 1). Another reason may be because the extent of fat mobilization reflected by plasma NEFA concentrations is much lower in first lactating cows as compared to cows with more parities. It is well established that increased utilization of NEFA for milk fat synthesis accounts not only for an increased total milk fat content but also for a higher proportion of long-chain fatty acids on the expense of short- and medium-chain fatty acids *de novo* synthesized from acetate or butyrate^13^. Hence, the mammary gland utilizes less acetate and as a consequence one might expect rising plasma acetate concentrations. Instead we observed lower plasma acetate concentrations in HM cows in week 5 p.p. which argues against our hypothesis proposed above - higher plasma NEFA concentrations would negatively affect metabolic acetate utilization - and led us to waive milk fatty acid profile analysis.

The lower plasma acetate concentrations in HM cows in week 5 p.p., but not at the other time points investigated, suggest that HM and LM cows differ in their acetate oxidation, acetate absorption or ruminal acetate production rates, and that these differences are specific for the early lactation period only. Acetate originates from carbohydrate fermentation and thus COX can be seen as an estimate for acetate oxidation. We found no differences in COX between groups in week 5 p.p., suggesting that metabolic acetate oxidation should be similar between HM and LM cows at this time. In addition, comparable COX between groups in week 5 p.p. also reflects the same level of DMI of HM and LM cows.

The absorption of acetate through the ruminal epithelium into the blood stream follows partly mass action diffusion and partly active protein mediated transporters^15^,^16^. The former depends largely on ruminal pH, capillary blood flow to the epithelium and the size of the epithelial surface area. Equally sized and aged cows fed the same diet are also expected to have a comparable rumen size and epithelial surface area. Also, the comparable rumen pH of HM and LM cows argues against a different acetate diffusion rate. Therefore, different active acetate transport rates in the two cow groups influenced by increased circulating NEFA concentrations are a credible feasibility, but this assumption remains to be investigated. The fact that we did not observe a significant relationship between acetate concentrations in plasma and in ruminal fluid enforces the idea that diminished active transport of acetate across the rumen wall contributes to reduced plasma acetate concentration in HM cows during early lactation. This assumption is supported by Dijkstra *et al*.^17^, who reported that ruminal concentrations of short chain fatty acids do not reflect their production rates. Other factors such as absorption rates, ruminal pH and the microbial populations account for this discrepancy^17^. Ultimately, we can only speculate whether the ruminal acetate production rate or the microbial composition differed temporally between HM and LM cows in week 5 p.p.

Interestingly we did not find a relationship between the ruminal acetate concentration and CH_4_ emission in the present study. This is in accordance with findings in sheep^18^, although other authors described a positive relationship between CH_4_ yield and ruminal acetate concentration or CH_4_ yield and acetate : propionate ratio^19^,^20^,^21^. However, the latter studies involved different diets that were fed to the animals, whereas all cows in the present study received the same diet at each time point investigated. Thus, a diet effect accounting for the rumen acetate to CH_4_ yield relationship described^19^,^20^,^21^ cannot be excluded.

As our initial hypothesis - plasma NEFA concentrations would negatively affect metabolic acetate utilization - could not be confirmed, we examined whether the extent of fat mobilisation is associative with different passage rates accounting for the different CH_4_ yield between in HM and LM cows. Increase in DMI usually increases the passage rate of the digesta through the gastro-intestinal tract, or vice versa decreases MRT, which in turn reduces feed digestibility and CH_4_ emission per unit feed ingested^22^,^23^,^24^. Conversely, CH_4_ within the gut has been shown to slow digesta transit and influence gut motility in dogs, guinea pigs and humans^25^. We found comparable MRT in the rumen-intestinal tract and feed digestibility in LM and HM cows, which is in line with the comparable DMI of both groups. The small differences in CH_4_ yield between cow groups in early lactation can therefore not be explained by MRT measured over the whole rumen-intestinal tract. Different studies have shown that the passage rate through the entire gastro-intestinal tract is not proportional to the reticulorumen passage of indigestible NDF in Holstein cows fed a corn silage based diet^26^,^27^. The precise measurement of the reticulorumen passage rate, however, requires the use of cannulated animals, which could not be realized in the present study. Goopy *et al*.^28^ described that sheep with a lower MRT of particulate and liquid matter in the rumen produced less CH_4_ despite the fact that their DMI was comparable with sheep possessing a higher ruminal MRT and CH_4_ yield. Sheep with lower CH_4_ yield had smaller rumen volumes^28^, however, general differences in the rumen size of HM and LM cows are unlikely because differences in CH_4_ yield between the two groups occurred only in week 5 p.p. and did not persist throughout whole lactation.

It might be that differences in CH_4_ yield between HM and LM cows in week 5 p.p. are owed to temporary differences in the MRT of the reticulorumen only, and not of the gastrointestinal tract as a whole, although we were not able to measure the reticulorumen-specific MRT in the present study. Dias *et al*.^29^ and Oshita *et al*.^30^ have shown that the fractional outflow rate of particulate matter from the reticulorumen was positively correlated with total chews, and that the decrease in particle size caused by chewing facilitates particle flow through the digestive tract. We did not evaluate chewing behavior of the animals, but because CH_4_ yield tended to differ only temporarily between HM and LM cows and both groups received the same diet, it seems unlikely that differences in chewing account for different MRT of the reticulorumen and therefore the statistical trend observed for CH_4_ yield in week 5 pp.

Possible causes for the approximately 10% different CH_4_ yield in HM and LM cows during early lactation may be temporary differences at the systemic side of the rumen-intestinal tract, e.g. differences in fat metabolism of the cows which affect the MRT of the reticulorumen and thus digestive processes. Several previous studies have shown that hormones related to fat metabolism and fat accretion can directly or indirectly influence gut motility. For example ghrelin and motilin stimulate gastric motility, accelerate gastric emptying and small intestinal transit time^31^,^32^. In an earlier study we have shown that HM cows have greater preprandial ghrelin concentrations than LM cows, and that this difference was particularly prominent during early lactation and less in late pregnancy^14^. This data indicates that HM cows have a higher motility at the proximal rumen-intestinal tract, conclusively reduced feed retention time in the rumen and therefore tend to have less CH_4_ yield in week 5 p.p. Moreover, 3^rd^ to 5^th^ lactating HM cows investigated in the earlier study^14^ had significantly less CH_4_ yield as compared to their LM counterparts in week 2 p.p. but not in week 6 a.p. (data not published yet) supporting the findings of the present study. However, whether administration of ghrelin directly affects CH_4_ emission remains to be examined in future studies. In contrast to ghrelin, intravenous infusion of cholecystokinin has been shown to depress frequency of reticular contractions and rumen motility^33^,^34^. Thus, lower CCK plasma concentrations in HM compared to LM cows in week 5 p.p. further underscores greater rumen motility which should promote an acceleration of ruminal passage rate in cows with greater fat mobilisation in early lactation.

Leptin, a hormone secreted by adipose tissue, interacts with the vagus nerve and the release of CCK and has a complex effect on motility of the gastrointestinal tract, e.g. by delaying gastric emptying and transit time of the ingesta through the small intestine^31^. It has been described that LM cows have lower plasma leptin concentrations than HM cows, but only before and not after parturition^35^. Thus, different CH_4_ yields observed as a trend between groups in week 5 p.p. can therefore not be explained by different plasma leptin concentrations.

Intravenous infusion of acetate did not modify gastric motility^36^ and conclusively it seems unlikely that differences in plasma acetate concentrations of HM and LM cows p.p. would affect motility of the gastrointestinal tract. The effect of circulating long-chain fatty acids on rumen or gut motility is not known, however, enterocytes are able to oxidize long-chain fatty acids to activate enteric neurons signaling to reduce feed intake^37^. Greater plasma NEFA concentrations and FOX rates in HM cows during early lactation argue against this mechanism because DMI is comparable to LM cows. This opens the question whether circulating NEFA may influence the motility of the gastrointestinal tract and consequently CH_4_ yield.

A further question to be answered is concerning the fate of the hydrogen in HM cows producing less CH_4_ in week 5 p.p. Possibly, hydrogen is increasingly transferred towards propionate although ruminal propionate concentrations were not different between groups. But keeping in mind that the total amount of propionate produced in the rumen exceeds the total amount of CH_4_ by far, conclusions on the hydrogen transfer towards propionate are difficult to draw. It is also conceivable that in HM cows less hydrogen is transferred to the butyrate producing pathway as indicated by the numerically lower ruminal butyrate concentrations in HM cows in week 5 p.p. and that this hydrogen is released via eructation.

## Materials and Methods

### Animals, feeding and milking

Twenty pregnant German Holstein heifers were kept in a free stall at the Leibniz-Institute for Farm Animal Biology (FBN), Dummerstorf, Germany and monitored until 291 DIM (SE ± 1.5) of their first lactation. 6 weeks prior to their expected calving date heifers were transferred to the straw bedded calving box and returned to the free stall on the first day after calving. All animals were treated in accordance with the guidelines for the use of animals as experimental subjects of the State Government in Mecklenburg-Western Pomerania. All experimental protocols were approved by the local animal ethics committee (Landesamt für Landwirtschaft, Lebensmittelsicherheit und Fischerei Mecklenburg-Vorpommern; approval No. 7221.1-1.-053/13).

Heifers had free access to water and the diet was offered as a standard ruminant total mixed ration (TMR) for ad libitum intake. 21 days before expected calving date animals were switched from the far-off to the close-up diet, and after parturition they received the lactation diet (Table 1). Individual daily feed intake was recorded as disappearance of feed from troughs connected to an electronic scale to which access was controlled by individual transponder (Roughage Intake Control, Insentec, Marknesse, The Netherlands). Based on the analysis of the individual TMR ration components, diet compositions for the far-off, close-up and lactation period were formulated and calculated according to the feeding standards of the German Society of Nutrition Physiology (GfE)^38^. An additional TMR sample was taken at the time of respiration chamber measurements (see below) for the determination of dry matter (DM) and diet composition. Analyses were conducted by the Landwirtschaftliche Untersuchungs- und Forschungsanstalt (LUFA) in Rostock, Germany. Chemical composition of TMR samples are shown in Table 1.

Feed energy was calculated according to Boguhn *et al*.^39^ and GfE^38^:


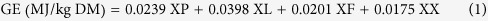



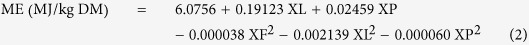



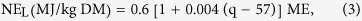


where GE is gross energy, ME is metabolizable energy, NE_L_ is net energy lactation, XP is crude protein, XL is crude fat, XF is crude fibre, XX is N free extracts and q = ME/GE x 100.

Body weight of the animals was measured automatically after milking and recorded as weekly means, and immediately before each indirect calorimetric measurement.

BFT measurements were conducted ultrasonographically (Titan Ultrasound System, SonoSite Inc., USA) from 1 until 14 weeks after calving at 14-day intervals. Additionally, BCS was determined at the same day on a 5 point scale according to Schroder and Staufenbiel^40^. Animals were milked twice daily at 04:30 h and 16:30 h and milk yield was recorded automatically. Milk samples from evening and morning milking were pooled and analyzed weekly by the Landeskontrollverband für Leistungs- und Qualitätsprüfung Mecklenburg-Vorpommern e.V. for milk composition. To calculate the ECM the following formula according to Reist *et al*.^41^ was used:


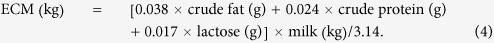


### Indirect Calorimetry

For CH_4_ measurement animals were transferred into open-circuit respiration chambers^42^ in week 4 a.p. and in week 5, 13 and 42 p.p. (SE ± 0.2 weeks). Animals were halter-trained and well adapted before measurements in the chamber, meaning habituation at least three times until the animal appeared relaxed and displayed regular behavior such as eating, ruminating and lying down. Within the chambers animals were kept in tie-stall at 15 °C, a dark–light cycle from 06:00 h to 19:00 h, milked twice daily if lactating and had access to fresh water. After an overnight stay allowing gas exchange equilibration, measurement of gas concentrations started at 07:00 h and lasted for 24 h. The CH_4_ and CO_2_ concentrations in the chamber were analyzed by infrared absorption and the O_2_ concentration was measured paramagnetically (SIDOR, SICK MAIHAK GmbH, Reute, Germany) as described recently by Derno *et al*.^42^. Air flow through the chambers was recorded with a differential-pressure type V-cone flow meter (McCrometer, Hemet, CA, USA). Fermentative CO_2_ (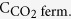) production was estimated from CH_4_ production according to Chwalibog *et al*.^43^:


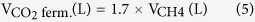


in which the factor 1.7 is constant for a variety of diet compositions^44^.

Metabolic CO_2_ (CO_2 metab._) was calculated as difference between total and fermentative CO_2_ production:


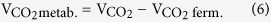


COX and FOX were calculated as described by Derno *et al*.^42^:


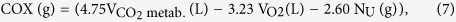



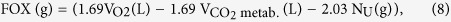


where N_U_ is urine N excretion. N_U_ was not measured and set to zero accepting an error of about 10%^43^ for both COX and FOX.

Feed was given twice at 07:30 h and 15:00 h. Feed intake in the chamber was determined by feed disappearance measured by using a scale connected to an electronic registration device. Data was collected every 6 min for 24 consecutive hours. Animals were milked in the chamber at 07:00 h and 16:30 h and the milk yield was recorded.

### Blood sampling

Animals were blood sampled at 07:00 h in the morning immediately before transferring into the respiration chambers by puncture of the Vena jugularis externa using BD Vacutainers containing potassium ethylenediaminetetraacetate (Greiner bio-one, Frickenhausen, Germany). Additional blood samples were taken weekly from week -3 to +12 relative to parturition (Supplemental Figure S1). Immediately after collection the vials were processed in a centrifuge at 4 °C for 20 minutes at 1,300 × g. Plasma was harvested and stored at −20 °C until analysis. Plasma concentrations of NEFA and BHBA were analyzed photometrically (Abx Pentra 400, Horiba ABX SAS, Montpellier, France) using kit no. 436-91995 for NEFA (Wako Chemicals GmbH, Neuss, Germany)and kit RB 1008 (Labor und Technik, Berlin, Germany) for BHBA. Plasma acetate concentrations were determined as chloroethyl ester derivative on a gas chromatography-flame ionization detector instrument (GC-FID, Series 2010, Shimadzu Corp., Kyoto, Japan) on a 25 m RTX-1701 column according to Kristensen *et al*.^45^. CCK was measured using a double antibody radioimmuno assay (Wizard 1470 Automatic Gamma Counter, Perkin Elmer, Waltham, USA) according to Relling and Reynolds^46^.

### Ruminal fluid

Ruminal fluid was collected immediately before animals were transferred into respiration chambers using an esophageal tube system attached to a vacuum pump. Rumen fluid was sieved (mash size 0.7–1.0 mm) and pH was determined using a glass electrode (Roth, Karlsruhe, Germany). The filtrate was centrifuged at 4 °C for 10 min at 4,000 g. Rumen fluid short-chain fatty acid concentrations were measured in the supernatant using a gas chromatograph (GC-FID, Series 17A, Shimadzu Corp., Kyoto, Japan), equipped with a 25 m FFAP column according to Ryan^47^.

### Gastrointestinal passage rate and digestibility

Analysis of gastrointestinal passage rate was performed in week 6 p.p. Cows were fed 15 g titanium dioxide (TiO_2_; pelleted with corn meal in a ratio of 1:2) twice daily for five days. Starting four days after begin of TiO_2_ application, feces samples (approx. 600 g) were taken twice daily at 08:30 h and 16:00 h for five days. Daily samples were pooled and stored at −20 °C until drying and analysis. Feces DM was determined after drying the samples at 65 °C for 72 h. TiO_2_ was analyzed according to the method described by Brandt and Allam^48^. For the calculation of the MRT of digesta we used the formula described by Voigt *et al*.^49^:


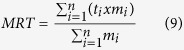


where m_i_ is the amount of TiO_2_ excreted at the *i*th sample and t_i_ is the time elapsed between dosing and the mid-point of the *i*th collection interval.

The Kjeldahl procedure was used to determine fecal N content with small modifications^50^. Fecal CP was calculated on an organic matter (OM) basis:


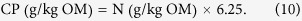


The OM digestibility of the ration was estimated using the formula of Lukas *et al*.^51^:


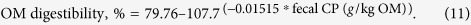


### Data handling and Statistics

Animals were grouped according to their plasma NEFA concentrations at the time of the respiration chamber measurements in early lactation (week 5 ± 0.2 p.p.) to the ten highest mobilizing (HM; plasma NEFA > 580 μmol/L) and the ten lowest mobilizing (LM; plasma NEFA < 580 μmol/L) cows. Weekly means of BFT and BCS were calculated from two consecutive measurements performed at 14 day intervals.

The data analysis was generated using SAS software, Version 9.3 for Windows, SAS Institute Inc., Cary, NC, USA. Variables BCS, BFT, DMI, CH_4_, milk, rumen fluid and plasma parameters were analyzed by repeated measurement ANOVA with the MIXED procedure of SAS/STAT software. The ANOVA models contained the fixed factors group (levels: HM, LM), time and the interaction group × time. The levels of the repeated variable time for milk data are weeks 5 p.p., 13 p.p., 42 p.p. and, for all other variables weeks 4 a.p., 5 p.p., 13 p.p., 42 p.p. Additionally, the levels of the repeated variable time for the variable DMI are weekly from 6 weeks a.p. to 14 weeks p.p, for the variable BW weekly from 4 weeks a.p. to 14 weeks p.p., and for the variables BCS, BFT, ECM are weekly from 1 week to 14 weeks p.p. Repeated measures on the same animal were taken into account by the REPEATED statement of the MIXED procedure and the type for the block diagonal residual covariance matrix was unstructured for the calculations with three or four time points and compound symmetry for the weekly calculations. Least-squares means (LSM) and their standard errors (SE) were computed for each fixed effect in the models, and all pairwise differences of LS-means were tested by the Tukey-Kramer procedure. The SLICE statement of the MIXED procedure was used for performing partitioned analyses of the LS-means for the interaction group × time. The MIXED procedure was also used to test the variable area under the curve (AUC) for the fixed factor group.

Linear relationships between variables NEFA, ruminal acetate and plasma acetate, CH_4_, CH_4_/DMI and CH_4_/NDF in week 5 p.p. were estimated and tested with the REG procedure of SAS/STAT software. Exponential relationships between variables CCK and CH_4_, CH_4_/DMI, CH_4_/NDF in week 5 p.p. were estimated and tested with the NLIN procedure of SAS/STAT software using the model formula y = a+b^(cx)^. Effects and differences were declared significant if *P* < 0.05 and trends as *P* < 0.1.

## Conclusions

We investigated the individual CH_4_ production of first lactating dairy cows to characterize the impact of body fat mobilization during the exceptional metabolic state in early lactation on ruminal fermentation characteristics. While ruminal acetate concentrations proved to be unrelated to levels of CH_4_ production, cows with high body fat mobilization and low plasma acetate concentrations tended to have lower CH_4_/DMI and CH_4_/NDF production rates in early lactation than less mobilizing cows. Lower plasma CCK concentrations in early lactation accounts for increased rumen motility and a faster digesta passage through the rumen (without affecting whole rumen-intestinal MRT) and therefore the lower CH_4_ yield of high mobilizing cows. The direct relationship between plasma CCK concentrations and CH_4_ yield as well as the inverse relationship between plasma NEFA concentrations and CH_4_ yield offers a new perspective on the interaction between host metabolism and rumen fermentation.

## Additional Information

**How to cite this article**: Bielak, A. *et al*. Body fat mobilization in early lactation influences methane production of dairy cows. *Sci. Rep.* **6**, 28135; doi: 10.1038/srep28135 (2016).

## Supplementary Material

Supplementary Information

## Figures and Tables

**Table 1:** Diet components and analysis of the total mixed ration (TMR) ingested during stays in respiration chamber.

**Component (g/kg DM)**	**Far-off**	**Close-up**	**Lactation**		
Grass silage	793	160	181		
Corn silage		432	325		
Grass hay	66	102	32		
Barley straw	133	63	38		
Corn kernels		45	70		
Grain mix			39		
Extracted soy meal		54	21		
Extracted canola seed meal		68	38		
Feed lime			4		
MF 2000 (concentrate)^1^		65	220		
Mineral 9522^2^			9		
Prenatal TR40^3^	11	11			
Chemical analysis		4 weeks a.p.	5 weeks p.p.	13 weeks p.p.	42 weeks p.p.
Crude ash	(g/kg DM)	86	69	68	73
Crude protein	(g/kg DM)	177	162	157	156
Crude fiber	(g/kg DM)	231	167	171	166
Crude fat	(g/kg DM)	21	31	30	32
Sugar	(g/kg DM)	10	26	29	21
Starch	(g/kg DM)	100	255	252	254
NDF	(g/kg DM)	501	345	347	371
ADF	(g/kg DM)	296	208	211	210
N free extracts	(g/kg DM)	407	494	494	494
ME	(MJ/kg DM)	9.6	11.3	11.2	11.3
NE_L_	(MJ/kg DM)	5.7	7.0	6.9	7.1

^1^MF 2000 (Vollkraft Mischfutterwerke GmbH, Güstrow, Germany): 7.1 MJ NE_L_/kg, 24% crude protein, 3.3% crude fat, 6.2% crude fiber, 8.4% crude ash, 0.7% calcium, 0.5% phosphorus, 0.65% sodium, vitamins A, D_3_, E, calcium iodate, cobalt carbonate, manganese oxide, zinc oxide, sodium selenite.

^2^Rinderstolz 9522 (Salvana GmbH, Sparrieshoop, Germany): 92% crude ash, 20% calcium, 5% phosphorus, 8% sodium, 6% magnesium, vitamins A, D_3_, E, copper sulfate, zinc oxide, manganese oxide, calcium iodate, sodium selenite, cobalt carbonate.

^3^Prenatal TR 40 (Salvana GmbH, Sparrieshoop, Germany): 75% crude ash, 4% calcium, 6% phosphorus, 15% magnesium, 8% sodium, vitamins A, D_3_, E, zinc oxide, manganese oxide, copper sulfate, calcium iodate, sodium selenite, cobalt carbonate.

## References

[b1] Demeyer D. I. & Van Nevel C. J. Methanogenesis and integrated part of carbohydrate fermentation and its control. in: McDonald I. W. & Warner A. C. I. (eds.) Digestion and Metabolism in the Ruminant. The University of New England Publishing Unit, Armidale, N.S.W., pp. 366–382, ISBN 0-85834-086-0 (1975).

[b2] Casanas M. A. A. . Methyl-coenzyme M reductase A as an indicator to estimate methane production from dairy cows. J. Dairy Sci. 98, 4074–4083, doi: 10.3168/jds.2015-9310 (2015). 25841964

[b3] Mohammed R., McGinn S. M. & Beauchemin K. A. Prediction of enteric methane output from milk fatty acid concentrations and rumen fermentation parameters in dairy cows fed sunflower, flax, or canola seeds. J. Dairy Sci. 94, 6057–6068, doi: 10.3168/jds.2011-4369 (2011). 22118093

[b4] Chilliard Y., Martin C., Rouel J. & Doreau M. Milk fatty acids in dairy cows fed whole crude linseed, extruded linseed, or linseed oil, and their relationship with methane output. J. Dairy Sci. 92, 5199–5211, doi: 10.3168/jds.2009-2375 (2009). 19762838

[b5] van Lingen H. J., Crompton L. A., Hendriks W. H., Reynolds C. K. & Dijkstra J. Meta-analysis of relationships between enteric methane yield and milk fatty acid profile in dairy cattle. J. Dairy Sci. 97, 7115–7132, doi: 10.3168/jds.2014-8268 (2014). 25218750

[b6] Johnson K. A. . The effect of oilseeds in diets of lactating cows on milk production and methane emissions. J. Dairy Sci. 85, 1509–1515 (2002). 12146483 10.3168/jds.S0022-0302(02)74220-3

[b7] Martin C., Rouel J., Jouany J. P., Doreau M. & Chilliard Y. Methane output and diet digestibility in response to feeding dairy cows crude linseed, extruded linseed, or linseed oil. J. Anim. Sci. 86, 2642–2650, doi: 10.2527/jas.2007-0774 (2008). 18469051

[b8] Ricci P., Rooke J. A., Nevison I. & Waterhouse A. Methane emissions from beef and dairy cattle: Quantifying the effect of physiological stage and diet characteristics. J. Anim. Sci. 91, 5379–5389, doi: 10.2527/jas.2013-6544 (2013). 24174549

[b9] Lerch S. . Rapeseed or linseed in dairy cow diets over 2 consecutive lactations: Effects on adipose fatty acid profile and carry-over effects on milk fat composition in subsequent early lactation. J. Dairy Sci. 98, 1005–1018, doi: 10.3168/jds.2014-8578 (2015). 25483201

[b10] Vanlierde A. . Hot topic: Innovative lactation-stage-dependent prediction of methane emissions from milk mid-infrared spectra. J Dairy Sci 98, doi: 10.3168/jds.2014-8436 (2015). 26026761

[b11] Park A. F. . Characterization of ruminal dynamics in Holstein dairy cows during the periparturient period. J. Anim. Physiol. Anim. Nutr. 95, 571–582, doi: 10.1111/j.1439-0396.2010.01085.x (2011). 21091551

[b12] Adewuyi A. A., Gruys E. & van Eerdenburg F. Non esterified fatty acids (NEFA) in dairy cattle. A review. Vet. Q. 27, 117–126 (2005). 16238111 10.1080/01652176.2005.9695192

[b13] Palmquist D. L. In Advanced Dairy Chemistry Volume 2 Lipids (eds Fox P. F. & McSweeney P. L. H.) Ch. 2, 43–92, ISBN 978-0-387-28813-0 (Springer, US, 2006).

[b14] Borner S. . Plasma ghrelin is positively associated with body fat, liver fat and milk fat content but not with feed intake of dairy cows after parturition. J. Endocrinol. 216, 217–229, doi: 10.1530/joe-12-0384 (2013). 23160961

[b15] Kendall P. E. & McLeay L. M. Excitatory effects of volatile fatty acids on the *in vitro* motility of the rumen of sheep. Res. Vet. Sci. 61, 1–6, doi: 10.1016/s0034-5288(96)90101-0 (1996). 8819185

[b16] Storm A. C., Kristensen N. B. & Hanigan M. D. A model of ruminal volatile fatty acid absorption kinetics and rumen epithelial blood flow in lactating Holstein cows. J. Dairy Sci. 95, 2919–2934, doi: 10.3168/jds.2011-4239 (2012). 22612930

[b17] Dijkstra J. Production and absorption of volatile fatty-acids in the rumen. Livestock Production Science 39, 61–69, doi: 10.1016/0301-6226(94)90154-6 (1994).

[b18] Robinson D. L., Goopy J. & Hegarty R. S. Can rumen methane production be predicted from volatile fatty acid concentrations? Animal Production Science 50, 630–636, doi: 10.1071/an09214 (2010).

[b19] Hassanat F. . Replacing alfalfa silage with corn silage in dairy cow diets: Effects on enteric methane production, ruminal fermentation, digestion, N balance, and milk production. J. Dairy Sci. 96, 4553–4567, doi: 10.3168/jds.2012-6480 (2013). 23684039

[b20] Benchaar C. . Effects of increasing amounts of corn dried distillers grains with solubles in dairy cow diets on methane production, ruminal fermentation, digestion, N balance, and milk production. J. Dairy Sci. 96, 2413–2427, doi: 10.3168/jds.2012-6037 (2013). 23462175

[b21] Lettat A., Hassanat F. & Benchaar C. Corn silage in dairy cow diets to reduce ruminal methanogenesis: Effects on the rumen metabolically active microbial communities. J. Dairy Sci. 96, 5237–5248, doi: 10.3168/jds.2012-6481 (2013). 23769352

[b22] Benchaar C., Rivest J., Pomar C. & Chiquette J. Prediction of methane production from dairy cows using existing mechanistic models and regression equations. J. Anim. Sci. 76, 617–627, doi: /1998.762617x (1998). 9498373 10.2527/1998.762617x

[b23] Okine E. K., Mathison G. W., Kaske M., Kennelly J. J. & Christopherson R. J. Current understanding of the role of the reticulum and reticulo-omasal orifice in the central of digesta passage from the ruminoreticulum of sheep and cattle. Canadian Journal of Animal Science 78, 15–21, doi: 10.4141/A97-021 (1998).

[b24] Sauvant D. & Noziere P. The quantification of the main digestive processes in ruminants: the equations involved in the renewed energy and protein feed evaluation systems. Inra Productions Animales 26, 327–346, doi: 10.1017/S1751731115002670 (2013). 26696120

[b25] Pimentel M. . Methane, a gas produced by enteric bacteria, slows intestinal transit and augments small intestinal contractile activity. Am. J. Physiol.-Gastroint. Liver Physiol. 290, G1089–G1095, doi: 10.1152/ajpgi.00574.2004 (2006). 16293652

[b26] Kramer M., Lund P. & Weisbjerg M. R. Rumen passage kinetics of forage- and concentrate-derived fiber in dairy cows. J. Dairy Sci. 96, 3163–3176, doi: 10.3168/jds.2012-6146 (2013). 23498017

[b27] Warner D., Dijkstra J., Hendriks W. H. & Pellikaan W. F. Passage kinetics of C-13-labeled corn silage components through the gastrointestinal tract of dairy cows. J. Dairy Sci. 96, 5844–5858, doi: 10.3168/jds.2013-6694 (2013). 23831103

[b28] Goopy J. P. . Low-methane yield sheep have smaller rumens and shorter rumen retention time. Br. J. Nutr. 111, 578–585, doi: 10.1017/s0007114513002936 (2014). 24103253

[b29] Dias R. S. . Relationships between chewing behavior, digestibility, and digesta passage kinetics in steers fed oat hay at restricted and ad libitum intakes. J. Anim. Sci. 89, 1873–1880, doi: 10.2527/jas.2010-3156 (2011). 21297056

[b30] Oshita T., Sudo K., Nonaka K., Kume S. & Ochiai K. The effect of feed regimen on chewing time, digesta passage rate and particle size distribution in Holstein non-lactating cows fed pasture ad libitum. Livest. Sci. 113, 243–250, doi: 10.1016/j.livsci.2007.04.001 (2008).

[b31] Yarandi S. S., Hebbar G., Sauer C. G., Cole C. R. & Ziegler T. R. Diverse roles of leptin in the gastrointestinal tract: Modulation of motility, absorption, growth, and inflammation. Nutrition 27, 269–275, doi: 10.1016/j.nut.2010.07.004 (2011). 20947298 PMC3066025

[b32] Muller T. D. & Tschop M. H. Ghrelin - a key pleiotropic hormone-regulating systemic energy metabolism. Endocr Dev 25, 91–100, doi: 10.1159/000346590 (2013). 23652395

[b33] Kermani R. Z. & Rezaiee A. The effects of intravenous cholecystokinin, secretin and pentagastrin on electromyographic activity of the rumen in sheep. Regul. Pept. 45, 371–377, doi: 10.1016/0167-0115(93)90363-d (1993). 8351402

[b34] Grovum W. L. Factors affecting the voluntary intake of food by sheep 3. The effect of intravenous infusions of gastrin, cholecystokinin and secretin on motility of the reticulo-rumen and intake Br. J. Nutr. 45, 183–201, doi: 10.1079/bjn19810091 (1981). 7470434

[b35] Pires J. A. A., Delavaud C., Faulconnier Y., Pomies D. & Chilliard Y. Effects of body condition score at calving on indicators of fat and protein mobilization of periparturient Holstein-Friesian cows. J. Dairy Sci. 96, 6423–6439, doi: 10.3168/jds.2013-6801 (2013). 23910547

[b36] Cuche G., Cuber J. C. & Malbert C. H. Ileal short-chain fatty acids inhibit gastric motility by a humoral pathway. Am. J. Physiol.-Gastroint. Liver Physiol. 279, G925–G930, WOS:000090062700011 (2000). 10.1152/ajpgi.2000.279.5.G925 11052989

[b37] Langhans W., Leitner C. & Arnold M. Dietary fat sensing via fatty acid oxidation in enterocytes: possible role in the control of eating. Am. J. Physiol.-Regul. Integr. Comp. Physiol. 300, R554–R565, doi: 10.1152/ajpregu.00610.2010 (2011). 21148477

[b38] GfE (German Society of Nutrition Physiology) Ausschuss für Bedarfsnormen der Gesellschaft für Ernährungsphysiologie. Empfehlungen zur Energie- und Nährstoffversorgung der Milchkühe und Aufzuchtrinder (Recommended energy and nutrient supply for dairy cows and growing cattle). Vol. 13, ISBN-13: 978-3769005912 (DLG-Verlag, 2004).

[b39] Boguhn J., Kluth H., Steinhöfel O., Peterhänsel M. & Rodehutscord M. Nutrient digestibility and prediction of metabolizable energy in total mixed rations for ruminants. Archives of Animal Nutrition 57, 253–266, doi: 10.1080/00039420310001594405 (2003). 14533865

[b40] Schroder U. J. & Staufenbiel R. Methods to determine body fat reserves in the dairy cow with special regard to ultrasonographic measurement of backfat thickness. J. Dairy Sci. 89, 1–14, doi: 10.3168/jds.S0022-0302(06)72064-1 (2006). 16357263

[b41] Reist M. . Concentrate feeding strategy in lactating dairy cows: Metabolic and endocrine changes with emphasis on leptin. J. Dairy Sci. 86, 1690–1706, doi: 10.3168/jds.S0022-0302(03)73755-2 (2003). 12778580

[b42] Derno M. . Short-term feed intake is regulated by macronutrient oxidation in lactating Holstein cows. J. Dairy Sci. 96, 971–980, doi: 10.3168/jds.2012-5727 (2013). 23219119

[b43] Chwalibog A., Jensen K. & Thorbek G. Oxidation of nutrients in bull calves treated with beta-adrenergic agonists. Archives of Animal Nutrition-Archiv Fur Tierernahrung 49, 255–261, ISSN: 0003-942X (1996). 10.1080/17450399609381888 8988312

[b44] Blummel M., Aiple K. P., Steingass H. & Becker K. A note on the stoichiometrical relationship of short chain fatty acid production and gas formation *in vitro* in feedstuffs of widely differing quality. Journal of Animal Physiology and Animal Nutrition-Zeitschrift Fur Tierphysiologie Tierernahrung Und Futtermittelkunde 81, 157–167, doi: 10.1046/j.1439-0396.1999.813205.x (1999).

[b45] Kristensen N. B., Pierzynowski S. G. & Danfaer A. Net portal appearance of volatile fatty acids in sheep intraruminally infused with mixtures of acetate, propionate, isobutyrate, butyrate, and valerate. J. Anim. Sci. 78, 1372–1379, doi: /2000.7851372x (2000). 10834594 10.2527/2000.7851372x

[b46] Relling A. E. & Reynolds C. K. Feeding rumen-inert fats differing in their degree of saturation decreases intake and increases plasma concentrations of gut peptides in lactating dairy cows. J. Dairy Sci. 90, 1506–1515, doi: 10.3168/jds.S0022-0302(07)71636-3 (2007). 17297124

[b47] Ryan J. P. Determination of volatile fatty-acids and some related compounds in ovine rumen fluid, urine, and blood-plasma, by gas-liquid-chromatography. Analytical Biochemistry 108, 374–384, doi: 10.1016/0003-2697(80)90602-8 (1980). 7457884

[b48] Poppe S. & Allam. Workshop zu Problemen der Verdauungsphysiologie beim Wiederkäuer (Rostock, 16.-17. 1. 1986). Arch. Anim. Nutr 37, 451–466, doi: 10.1080/17450398709425369 (1987). 3454629

[b49] Voigt J., Jentsch W., Kuhla S., Matthes H. D. & Derno M. Rumen fermentation and retention time of the digests in growing cattle of the breeds Black-White Dairy Cattle, Galloway, and Highland. Arch. Tierz.-Arch. Anim. Breed. 43, 609–620, WOS:000165330200007 (2000).

[b50] Glindemann T., Tas B. M., Wang C., Alvers S. & Susenbeth A. Evaluation of titanium dioxide as an inert marker for estimating faecal excretion in grazing sheep. Anim. Feed Sci. Technol. 152, 186–197, doi: 10.1016/j.anifeedsci.2009.04.010 (2009).

[b51] Lukas M., Sudekum K. H., Rave G., Friedel K. & Susenbeth A. Relationship between fecal crude protein concentration and diet organic matter digestibility in cattle. J. Anim. Sci. 83, 1332–1344, doi: /2005.8361332x (2005). 15890810 10.2527/2005.8361332x

