# Supplementary material for: Body fat mobilization in early lactation influences methane production of dairy cows
Source: Sci Rep. 2016 Jun 16;6:28135. doi: 10.1038/srep28135 (PMC4910095; doi:10.1038/srep28135)
Supplement: Supplementary Information [file srep28135-s1.pdf]

Body fat mobilization in early lactation influences methane production of dairy cows

A. Bielak, M. Derno, A. Tuchscherer, H.M. Hammon, A. Susenbeth, B. Kuhla

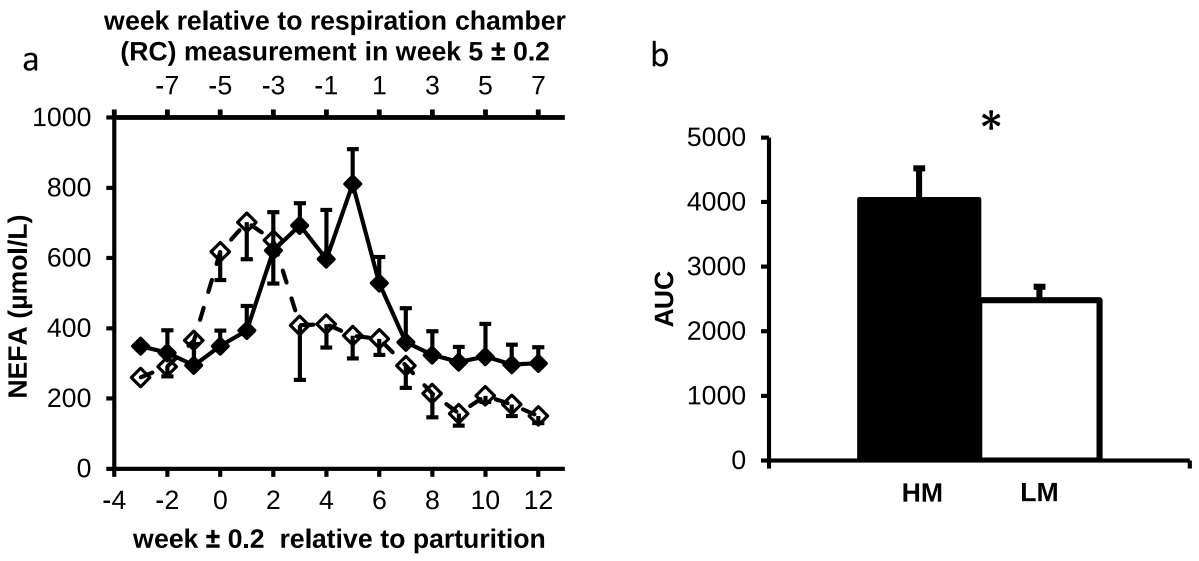

Supplemental Figure S1.
a: Plasma NEFA of high (♦, solid line; n = 10) and low mobilizing (◊, dashed line; n = 10) cows in relation to the respiration chamber measurement in early lactation, and in relation to weeks relative to parturition. ANOVA calculated time×group interaction $P < 0.001$.
b: Area under the curve (AUC) of the graph in Figure 1a calculated for high (■; n = 10) and low mobilizing (□; n = 10) cows for the time period 2 weeks before until 6 weeks after the respiration chamber measurement in week 5 ± 0.2 post partum. $P = 0.007$
Data in bar chart is shown as LSM ± SE.

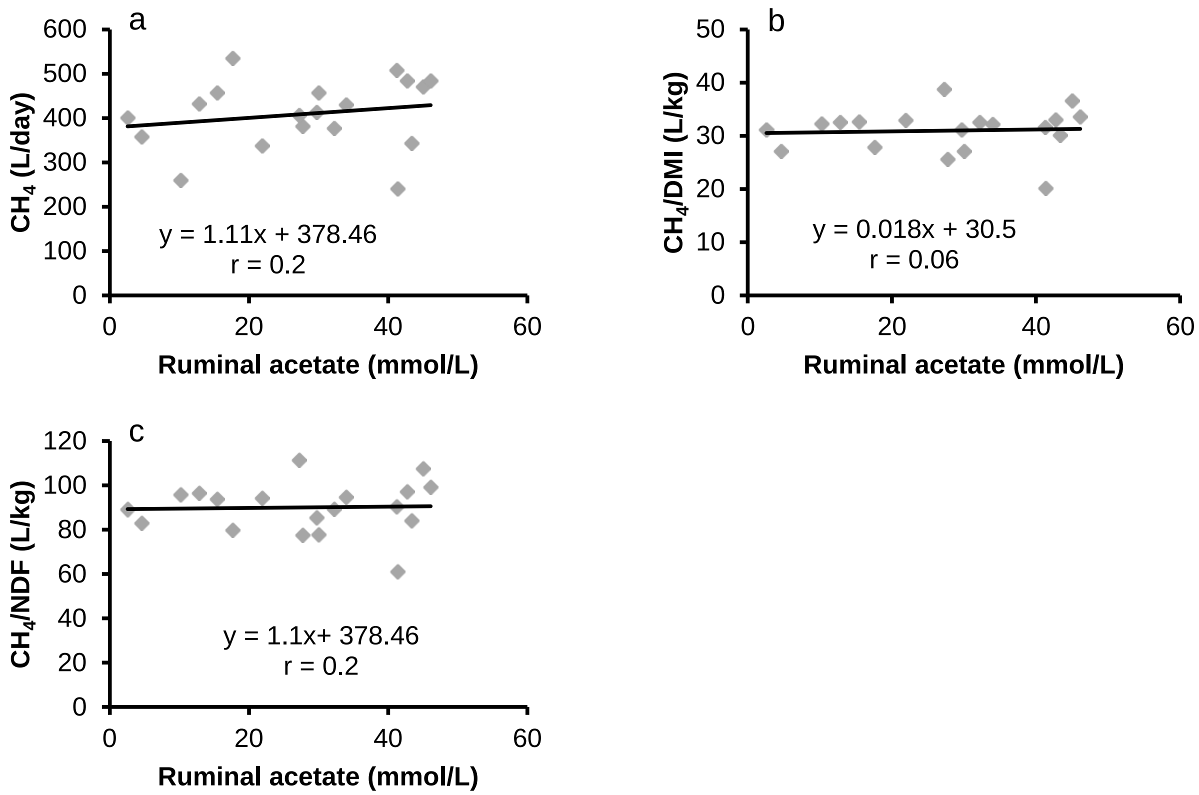

Supplemental Figure S2. Linear regression between ruminal acetate concentration and daily CH4 production either expressed as L/d, L/kg DMI, or L/kg NDF, respectively (n=19).

a: slope P = 0.42

b: slope P = 0.80

c: slope P = 0.42

Supplemental Table S1. Animal and performance data of high mobilizing (HM) and low mobilizing (LM) cows during stays in respiration chamber

	4 weeks a.p.			5 weeks p.p.			13 weeks p.p.			42 weeks p.p.			Statistics ANOVA, *P* values		
Group	HM	LM	STE	HM	LM	STE	HM	LM	STE	HM	LM	STE	Time	Group	Time×Group
Body weight (kg)	626.4	631.9	±11.7	568.7	552.9	±16.1	557.7	553.8	±15.8	627.7	636.3	±20.0	< 0.001	0.88	0.65
DMI (kg)	6.5	7.6	±0.7	13.5	12.9	±0.8	14.2	14.8	±0.6	15.8	14.6	±0.7	< 0.001	0.97	0.09
BCS	-	-		2.8	2.7	±0.2	2.7	2.7	±0.2	-	-		0.37	0.71	0.44
BFT (cm)	-	-		1.5†	1.2†	±0.1	1.0	0.9	±0.1	-	-		< 0.001	0.21	0.03
Milk yield (kg/day)	-	-		27.03	26.22	±1.37	28.16	26.81	±1.16	23.49	22.43	±1.42	< 0.001	0.47	0.94
Milk fat (%)	-	-		4.69	4.95	±0.37	3.72*	4.34*	±0.13	4.18	4.71	±0.23	0.003	0.06	0.74
Milk protein (%)	-	-		3.07	3.09	±0.08	3.15	3.36	±0.05	3.78	3.85	±0.08	< 0.001	0.17	0.09
Milk lactose (%)	-	-		4.91	4.94	±0.06	5.00	4.99	±0.05	4.94	4.81	±0.05	0.002	0.66	0.06
ECM (kg/day)	-	-		28.81	28.70	±1.48	27.05	28.14	±1.18	24.68	25.18	±1.36	0.002	0.79	0.80

† P = 0.07 Tukey-Kramer

* P = 0.03 Tukey-Kramer
